# Supplementary material for: Identification of photoperiod-induced specific miRNAs in the adrenal glands of Sunite sheep (Ovis aries)
Source: Front Vet Sci. 2022 Jul 22;9:888207. doi: 10.3389/fvets.2022.888207 (PMC9354845; doi:10.3389/fvets.2022.888207)
Supplement: Supplementary file 1 [file Data_Sheet_1.ZIP › Supplementary file/miRDeep2 file.zip!/miRDeep2 file/Novel_Structure/LP42A3/Novel_411.pdf]

| | | |
|---|---|---|
| Provisional ID | : | NC_019464.2_10057 |
| Score total | : | 0.7 |
| Score for star read(s) | : | -1.3 |
| Score for read counts | : | 0 |
| Score for mfe | : | 0.4 |
| Score for randfold | : | 1.6 |
| Score for cons. seed | : | |
| Total read count | : | 37 |
| Mature read count | : | 37 |
| Loop read count | : | 0 |
| Star read count | : | 0 |

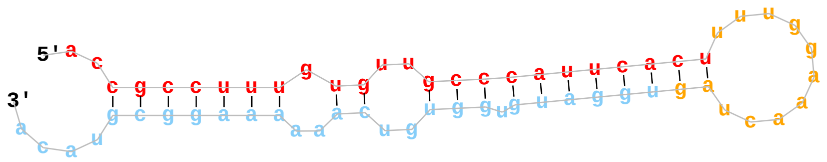


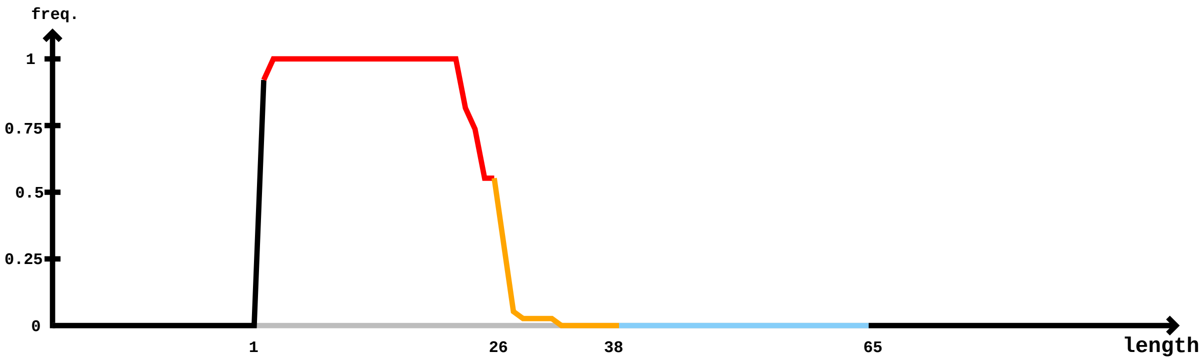


**Mature** **Star**

```
5'- auuccuuaugcgguuuucuuaccgccuuuguguugcccauucacuuuuggaaacuaguggauguggugucaaaaaaggcguacacaaaacacuuucgcagcuuuuuuuccugccc  -3'   exp
    ............(((((.....(((((((.((..(((((((((((..........)))))))).)))..))..)))))))....))))).....((((.........))))..  reads   mm   sample
    ....................accgccuuuguguugcccauu..........................................................................  7       0    seq
    ....................accgccuuuguguugcccauuc.........................................................................  3       0    seq
    ....................accgccuuuguguugcccauuca........................................................................  7       0    seq
    ....................accgccuuuguguugcccauucacu......................................................................  10      0    seq
    ....................accgccuuuguguugcccauucacuu.....................................................................  6       0    seq
    ....................accgccuuuguguugcccauucacuuu....................................................................  1       0    seq
    ....................accgccuuuguguugcccauucacuuuugga................................................................  1       0    seq
    .....................ccgccuuuguguugcccauucacu......................................................................  3       0    seq
```
